# Agranulocytosis – Sequelae of Chronic Cocaine Use: Case Series and Literature Review

**DOI:** 10.7759/cureus.1221

**Published:** 2017-05-03

**Authors:** Roma Srivastava, Maira Rizwan, Muhammad Omer Jamil, Palaniandy Kogulan, Donna Salzman

**Affiliations:** 1 Hematology/Oncology, The Joan C Edwards College of Medicine of Marshall University, Huntington, WV; 2 Internal Medicine, Dow Medical College, Karachi, Pakistan; 3 Covenant Healthcare, Central Michigan University; 4 Blood and Marrow Transplantation and Cell Therapy Program, Division of Hematology and Oncology, University of Alabama at Birmingham

**Keywords:** cocaine, agranulocytosis, levamisole, typhlitis

## Abstract

Agranulocytosis is a rare condition with a reported incidence ranging from one to five cases per million population per year. Most commonly, agranulocytosis is secondary to chemotherapeutic agents, however, other medications have also been associated with it. An immune mediated destruction of circulating granulocytes and/or granulocyte precursors secondary to drug-dependent or drug-induced antibodies is the postulated mechanism. Agranulocytosis has also been reported secondary to recreational drug use. Cocaine is one of the most commonly used recreational drugs and is often laced with Levamisole to enhance its psychostimulatory properties. Levamisole is an immune modulator and can cause bone marrow suppression. It can be detected in urine by gas chromatography-mass spectrometry.

We report two cases of recurrent agranulocytosis in non-oncology patients secondary to chronic cocaine abuse, who were treated with granulocyte colony-stimulating factor (GCSF) and broad spectrum antibiotics without sustained response.

The high prevalence of cocaine use continues to be a serious public health concern. This case series discusses the association of adulterated cocaine as an etiology of unexplained neutropenia and highlights the diverse clinical complications of chronic cocaine abuse. Currently, the available literature is reviewed.

## Introduction

Drug-induced neutropenia/agranulocytosis is a well-known complication of chemotherapeutic agents. Neutropenia is defined as an absolute neutrophil count (ANC) <1500/microL. The risk of fatal infection increases when the ANC falls to less than 1000/microL, and is most clinically significant when the ANC is <500/microL. Agranulocytosis/neutropenia is an extremely rare hematological condition in a non-oncology world. The annual incidence of agranulocytosis ranges from 1.1 to 4.9 cases per million populations per year [1].

Cocaine is the second most common drug of abuse in the United States and is often laced or adulterated with certain substances to increase the bulk of the drug and its euphoric effect. Levamisole, originally developed as an antirheumatologic agent and now used as a veterinary anthelmintic, is one such adulterant which is commonly used as a “cutting agent.” In 2011, U.S. Drug Enforcement Administration (DEA) estimated that 82% of seized cocaine contained levamisole [2]. It is known to be associated with agranulocytosis leading to a fatal outcome. We present two cases of agranulocytosis secondary to chronic cocaine use.

## Case presentation

### Case 1

A 43-year-old male was admitted in the intensive care unit with fever, tachycardia, and severe right lower abdominal pain. He was diagnosed with severe sepsis with possible intra-abdominal source and was treated with aggressive hydration, broad spectrum antibiotics, bowel rest, and analgesics. The computed tomography (CT) scan of the abdomen revealed large inflammatory mass involving the cecum and ascending colon with extensive pericolonic inflammation and mesenteric lymphadenopathy. The laboratory data revealed leukopenia with white blood cell (WBC) 0.33 K/cubic milliliters and absolute granulocytes (ANC) of 100 cells/milliliters. He developed bowel perforation requiring emergent right hemicolectomy with ileostomy. The histopathology of the resected cecum revealed bowel infarct with abundant bacterial and fungal elements infiltrating the bowel wall consistent with the diagnosis of necrotizing enterocolitis.

Review of his chart revealed that he has had multiple admissions in the last two years for recurrent neutropenia, each time treated with granulocyte colony-stimulating factors (GCSF). Bone marrow biopsy done as a part of the workup for neutropenia revealed hypocellular marrow with a marked decrease in the myeloid lineage. He admitted to chronic cocaine use and the urine analysis revealed the presence of cocaine and levamisole, which explained the etiology of agranulocytosis. He was referred to the drug rehabilitation program and remained asymptomatic thereafter.

### Case 2

A 49-year-old female presented to our transplant clinic for evaluation for allogeneic bone stem cell transplant for chronic neutropenia. Her neutropenia was first recognized about five years ago when she was admitted to the hospital for pneumonia and was found to have an absolute neutrophil count of 468 cells/ml. She reported frequent skin and respiratory tract infections over the past year. During the course of her illness, she was treated with multiple broad spectrum antibiotics. An exhaustive workup for neutropenia was done including bone marrow biopsy which revealed hypocellular marrow with a marked decrease in the myeloid lineage. She was treated with filgrastim injections with a transient improvement in her neutrophil count (ANC). She was followed by the hematology service and continued to be neutropenic with ANC ranging 20-800 cells/ml over the course of five years. She was treated with GCSF, prednisone, cyclosporine, anti-thymocyte globulin, and alemtuzumab at different times considering an autoimmune etiology without any sustained response. Over these years of follow-up and workup, she tested positive for HLA-B27 genotype and c-ANCA. She admitted to using cocaine over the past five years, successfully underwent drug rehabilitation program, and discontinued cocaine use without evidence of resumption. Finally, bone marrow transplant was not deemed necessary and she continued to follow up in the hematology clinic.

## Discussion

Levamisole is a synthetic imidazothiazole derivative used for its immunomodulatory properties. Developed in the 1970s it has been used as a disease modifying agent in rheumatoid arthritis. In 1990, it was approved by the Food and Drug Administration (FDA) for the treatment of colon cancer in conjunction with 5-flourouracil. However, in 2000 and 2003 levamisole was taken off the market in the United States and Canada due to reports of agranulocytosis. Currently, it is being used as an antihelminthic agent in veterinary medicine [2-3].

The exact purpose of levamisole use as an adulterant for cocaine is unclear and multiple theories exist. It is inexpensive, colorless, tasteless, and adds bulk to cocaine. It prolongs the euphoric effects of cocaine by potentiating the nicotinic acetylcholinergic effects. Metabolism of levamisole to aminorex and related compounds, specifically 4-methylaminorex, or “ice,” has high abuse potential because of their amphetamine-like pharmacological activities [3].

 It acts as an immune modulator and immune enhancer by enhancing the dendritic cell maturation leading to increased production of cytokines such as IL-12 and IL-10 to help T-cell activation. IL-12 plays a central role as a link between the innate and adaptive immune systems, promote T helper immune responses, induce the production of large amounts of IFN-γ, and activate T and natural killer (NK) cells. Levamisole-induced production of IL-12 is mediated through Toll-like receptor (TLR)-2 [4]. The adulterant can be detected in urine by gas chromatography-mass spectrometry [5].

Significant toxicities of levamisole include agranulocytosis, leukocytoclastic vasculitis, retiform purpura, and seizures [6].

Reports of agranulocytosis with the use of cocaine adulterated with levamisole were first reported in 2008. Since then several case reports of levamisole toxicity in patients using cocaine have been published [2]. Diagnosis typically relies on the history of cocaine abuse.

The mechanism of agranulocytosis caused by levamisole is not completely understood. It was initially reported as an idiosyncratic reaction in patients treated with levamisole for rheumatoid arthritis. Later, patients developing neutropenia with levamisole were shown to be positive for HLA-B27 genotype [7]. Agranulocytosis also more commonly occurs in females and in smokers of crack cocaine. The supposition of the immune-mediated mechanism of levamisole-induced agranulocytosis is also supported by the fact that it has been shown to induce leukocyte agglutinating and lymph cytotoxic antibodies or both [8].

Typhlitis or neutropenic enterocolitis is a rare condition and is clinically defined by the triad of neutropenia, abdominal pain, and fever [9]. Diagnosis is further supported by imaging. The true incidence of typhlitis is not known. In a 2007 report, it was diagnosed in 3.5 percent of 317 episodes of severe neutropenia among individuals more than 16 years of age [10]. It can complicate the treatment of patients with solid tumors and granulocytopenia from any cause. Though cocaine-induced neutropenia has been reported in the literature, but cocaine-induced typhlitis is an extremely rare occurrence.

In our case series, the urine specimen of the first patient tested positive for levamisole. He met the clinical, radiological, and pathological criteria of typhlitis, which was attributed to the tainted cocaine use. The second patient had a very high titer of c-ANCA, was positive for HLA-B27 genotype, lending further support towards levamisole as the underlying etiology of her agranulocytosis.

The primary treatment of agranulocytosis is usually supportive and most patients recover within 5-10 days of cessation of cocaine use. Patients should avoid future exposure to cocaine as it has shown some delayed hypersensitivity-like features where patients develop more severe agranulocytosis with each subsequent exposure.

## Conclusions

The high prevalence of levamisole-adulterated cocaine and potential toxicity in cocaine users is a serious public health concern. It is unclear why levamisole causes agranulocytosis in some patients but not in others and also what length of exposure is necessary to cause agranulocytosis. Clinical complications are diverse, under-reported, and the treatment success hinges on the high index of suspicion, early diagnosis, and cessation of cocaine use.
